# Optimizing infection management after cardiac arrest: addressing diagnostic uncertainty and therapeutic dilemmas—a narrative review

**DOI:** 10.1186/s40560-026-00859-6

**Published:** 2026-01-28

**Authors:** Jun Hagiwara, Keitaro Yoshioka, Kanako Ito-Hagiwara, Yusuke Endo, Daniel Jafari, Daniel M. Rolston, Cyrus E. Kuschner, Lance B. Becker, Kei Hayashida

**Affiliations:** 1 Laboratory for Critical Care Physiology, Feinstein Institutes for Medical Research, Northwell Health System, Manhasset, NY USA; 2 Department of Emergency Medicine, Donald and Barbara Zucker School of Medicine at Hofstra/Northwell, Hempstead, NY USA

**Keywords:** Post-cardiac arrest syndrome, Infection incidence, Targeted temperature management, Pneumonia, Biomarkers, Antimicrobial stewardship

## Abstract

Infections are frequent after cardiac arrest and materially affect post-ICU care and outcomes. Diagnostic uncertainty is heightened by post-cardiac arrest syndrome (PCAS)—hypoxic–ischemic brain injury, myocardial dysfunction, systemic ischemia–reperfusion injury, and immune dysregulation—and by sedation and targeted temperature management (TTM), which can mask clinical signs and modulate host defenses. Pneumonia predominates; bloodstream infection and intra-abdominal or hepatobiliary infections are under-recognized, especially in device-dependent or extracorporeal membrane oxygenation (ECMO)-treated patients. Conventional biomarkers such as C-reactive protein and procalcitonin show reduced infection specificity early after return of spontaneous circulation; therefore, single timepoint cutoffs are unreliable, and serial trajectories interpreted with clinical examination, microbiology, and imaging are preferred. Risk scores (e.g., Sequential Organ Failure Assessment [SOFA], Clinical Pulmonary Infection Score [CPIS]) may support stratification but are insufficient for definitive diagnosis. Observational cohorts report higher pneumonia rates with TTM, and temperature control can blunt fever and leukocytosis and alter cytokine and biomarker kinetics, complicating timely recognition. Prevention should emphasize protocolized bundles, including hand hygiene and asepsis, head-of-bed elevation, structured oral care per local policy, minimization of sedation with spontaneous breathing trials, and early removal of unnecessary devices, within an antimicrobial stewardship framework that supports early de-escalation once cultures and trajectories clarify etiology. Procalcitonin-guided early discontinuation reduces antibiotic exposure in general critical-care populations; in PCAS, use should prioritize serial trends integrated with clinical context rather than single thresholds. No fixed algorithm is prescribed; instead, practical considerations are presented to guide diagnostic practice, emphasizing early microbiological sampling and imaging, integration of serial biomarker trajectories with clinical assessment, and timely de-escalation as the clinical picture clarifies, without endorsing single-test cutoffs. Priorities include quantifying infection-attributable morbidity and mortality; developing and validating PCAS-specific biomarkers and composite decision tools, including electronic health record-based early warning models; evaluating short post-intubation prophylaxis in selected high-risk patients; optimizing TTM parameters (target temperature, duration, rewarming rate); and systematically characterizing under-recognized infections. A protocol-driven, multimodal program that integrates prevention, standardized diagnostics, and stewardship is required to deliver timely, appropriate therapy and improve outcomes after cardiac arrest.

**Graphical Abstract:**

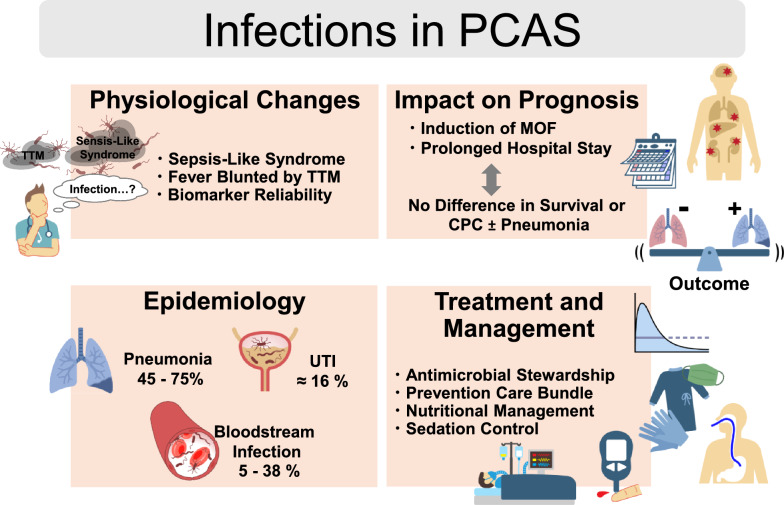

## Introduction

Cardiac arrest is a critical emergency marked by the abrupt cessation of both circulation and respiration, resulting in extremely high mortality without prompt resuscitative efforts [1, 2]. In the United States, approximately 350,000 cases of out-of-hospital cardiac arrest (OHCA) and 290,000 cases of in-hospital cardiac arrest (IHCA) occur annually [3, 4]. In Japan, more than 120,000 OHCA cases occur annually, and despite incremental improvements, the 1-month survival rate remains approximately 6% [5]. In addition, a nationwide inpatient database study estimated that IHCA cases number approximately 87,000 per year [6]. Survival following OHCA remains low, highly dependent on the efficiency of emergency medical services, the timeliness of bystander intervention, and post-arrest care [7]. In contrast, IHCA typically occurs in patients with significant comorbidities. Although early medical intervention is available, outcomes are often poor due to underlying illness severity [1, 8]. Despite therapeutic progress, patients after return of spontaneous circulation (ROSC) often develop post-cardiac arrest syndrome (PCAS), a multifactorial condition involving hypoxic–ischemic brain injury, myocardial dysfunction, systemic ischemia–reperfusion injury, and dysregulated immune responses. This immune dysfunction predisposes patients to secondary infections, including pneumonia, urinary tract infection (UTI), catheter-associated urinary tract infections (CAUTIs), and catheter-related bloodstream infections (CRBSIs) [9, 10]. These infections frequently progress to sepsis in the post-cardiac arrest setting. Notably, both the resuscitation process and subsequent post-arrest intensive care can impair cellular immunity, thereby exacerbating the risk of infection and multi-organ dysfunction [11, 12].

Targeted temperature management (TTM), widely implemented for its neuroprotective benefits, may adversely suppress immune function and obscure clinical signs of infection, such as fever, thereby complicating early diagnosis [13–15]. Pneumonia is the most common infectious complication after cardiac arrest and often involves a mix of aspiration-related pathogens as well as nosocomial or multidrug-resistant organisms, particularly in patients requiring invasive devices in the intensive care unit (ICU) [10]. Given these challenges, optimizing infection prevention, early detection, and appropriate treatment is essential to improve survival and neurological outcomes in patients with PCAS [16, 17].

However, despite increasing recognition of these issues, there remains a lack of consensus and evidence-based guidance on infection management specific to PCAS. In particular, the impact of immune dysregulation, the role of TTM, and the best practices for diagnosis and treatment in this context remain underexplored.

Therefore, this review provides an updated synthesis of the current knowledge regarding infection management in patients with PCAS. It addresses the following: (1) the epidemiology and major types of infection after cardiac arrest, including pneumonia, UTIs, bloodstream infections (BSIs), and intra-abdominal infections; (2) the impact of these infections on clinical outcomes, such as mortality, neurological recovery, and ICU resource utilization; (3) the pathophysiological mechanisms and diagnostic challenges associated with immune dysregulation, ischemia–reperfusion injury, sedation practices, and targeted temperature management; and (4) current strategies for prevention and management, including antimicrobial therapy, nonpharmacologic approaches, and developing integrative care pathways.

Through this structure, the review aims to provide clinicians and researchers with practical and comprehensive insights that can optimize infection control and improve outcomes for patients with PCAS.

## Methodology

This review was conducted as a narrative synthesis of the current literature to comprehensively examine the epidemiology, pathophysiology, diagnostic challenges, and management strategies related to infections in patients with PCAS. Given the complex interplay between immune dysfunction, TTM, and the frequent use of invasive interventions, a narrative review was deemed the most appropriate approach to capture the breadth and nuance of existing evidence across basic science, clinical studies, and expert recommendations.

A structured search of the PubMed database was performed to identify relevant English-language publications published from database inception through April 2025. The search strategy included both Medical Subject Headings (MeSH) and free-text terms. Keywords were combined using Boolean operators (AND, OR) and included “cardiac arrest,” “post-cardiac arrest syndrome,” “infection,” “sepsis,” “pneumonia,” “bacteremia,” “urinary tract infection,” “ventilator-associated pneumonia,” and “intra-abdominal infection.” The search was conducted iteratively to refine results and ensure inclusion of both foundational and recently emerging literature.

Studies were considered eligible if they met the following inclusion criteria: (1) peer-reviewed original research articles, systematic reviews, or clinical guidelines, (2) focus on adult patients with PCAS, and (3) evaluation of infections, either as primary or secondary outcomes, following ROSC. Exclusion criteria included: (1) studies limited to pediatric populations, (2) non-peer-reviewed materials, such as editorials or letters, (3) case reports and conference abstracts lacking full data, and (4) studies focused solely on pre-arrest infections or chronic infections unrelated to ICU care.

To enhance comprehensiveness, the reference lists of key articles were manually reviewed for additional relevant studies. Particular attention was given to large multicenter cohorts, observational studies involving TTM and trials evaluating infection prevention or antimicrobial therapy in post-cardiac arrest populations. Guidelines from major societies—such as the American Heart Association, European Resuscitation Council, and the Infectious Diseases Society of America—were also reviewed and incorporated when relevant.

This review does not include a formal risk-of-bias assessment or meta-analysis, consistent with the methodology of narrative synthesis. While this approach limits quantitative comparison, it allows for contextual integration of findings across heterogeneous study designs. Emphasis was placed on clinically meaningful insights, trends across populations, and practical implications for intensive care clinicians managing PCAS.

Through this method, the review aims to identify common patterns, highlight areas of uncertainty, and propose future research directions that can inform integrated infection control strategies in the post-cardiac arrest setting.

In this review, the term “infection rate” is used to indicate the proportion of patients who developed infection among all patients achieving ROSC, unless otherwise specified. Some cited studies use the term “rate” or “incidence” according to their original definitions; in such cases, we preserved the terminology used in the original publication. When the proportion was not explicitly reported, we calculated it using the available data from the study.

Although recent guidelines broadly use the term TTM to describe controlled temperature regulation between 32 and 36 °C, most of the historical evidence on infection risk after cardiac arrest is based on studies employing therapeutic hypothermia (TH, 32–34 °C). Therefore, in this review, “TTM” is used when referring to temperature management in general, whereas “TH” is used when describing findings derived specifically from hypothermia protocols, which constitute the primary evidence base regarding infection-related outcomes.

## Epidemiology and types of infection in PCAS

### Overview of infection epidemiology

Infections are common and clinically significant complications in patients with PCAS. Cohort studies report that 45–70% of patients admitted to the ICU after cardiac arrest develop at least one infection during their post-resuscitation course [16, 18]. Among these, pneumonia is the most prevalent, followed by UTI and BSI. These patterns are summarized in Table 1**,** which outlines the proportion of major infection types reported across key studies and highlights the consistently high burden of pneumonia in PCAS.

**Table 1:** Summary of infection proportion and TTM implementation in post-cardiac arrest patients

Study	Arrest type	TTM performed	Type of infection	Infection proportion (%)	Onset window (from admission)
Rello et al. 1995 [23]	NR	NR	Pneumonia	24	NR
Gajic et al. 2004 [31]	NR	NR	Pneumonia	30	NR
			BSI	9	NR
			UTI	7	NR
Tsai et al. 2005 [20] ^a^	OHCA	NR	Pneumonia	61	≤ 7 days
			BSI	13	≤ 7 days
			UTI	8	≤ 7 days
			IAI	3	≤ 7 days
Nielsen et al. 2011 [21] ^b^	OHCA	100%	Pneumonia	48	NR
Mongardon et al. 2011 [16]	OHCA	79%	Pneumonia	75	≤ 5 and > 5 days
			BSI	8.3	NR
			UTI	1.0	NR
			IAI	1.2	NR
Perbet et al. 2011 [15]	OHCA	78%	Pneumonia	65	≤ 3 days
Pabst et al. 2013 [49] ^b^	OHCA	100%	Pneumonia	52	≤ 4 days
Woo et al. 2014 [22] ^b^	OHCA	100%	Pneumonia	48	≤ 7 days
Coba et al. 2014 [32] ^c^	OHCA	NR	BSI	38	NR
Mortensen et al. 2020 [18] ^d^	OHCA	73%	Pneumonia	43	NR
			BSI	5	NR
			UTI	6	NR
	IHCA	30%	Pneumonia	48	NR
			BSI	5	NR
			UTI	16	NR
Baekgaard et al. 2020 [118]	OHCA	35%	Pneumonia	53	≤ 4 days

TTM: Targeted temperature management; NR: not reported; UTI: urinary tract infection; BSI: bloodstream infection; IAI: intra-abdominal infection; OHCA: out-of-hospital cardiac arrest; IHCA: in-hospital cardiac arrest

^a^ This study mentioned that, of the 117 patients included, 16 had an infection prior to cardiac arrest

^b^ These studies included only patients who received TTM

^c^ Only patients who underwent blood culture were included

^d^ Patients with documented infection prior to arrest were excluded

*Note* “Infection Proportion (%)” represents the percentage of patients who developed infection during the observation period reported in each study. The length and timing of observation varied, and several studies did not specify an exact onset window (NR). Therefore, values reflect proportions rather than incidence rates, and in some studies may include infections present prior to cardiac arrest unless otherwise indicated by footnotes

Mechanisms differ between arrest settings. OHCA patients are prone to aspiration-related pneumonia due to impaired consciousness during or prior to arrest, whereas IHCA patients, who have greater exposure to indwelling devices and longer pre-arrest hospitalization, develop more nosocomial infections, such as ventilator-associated pneumonia (VAP), CAUTI, and CRBSI [10, 19]. In a multicenter analysis, Mortensen et al. reported higher overall infection rates in IHCA than OHCA patients (66% vs. 53%), with pneumonia as the leading diagnosis in both groups [18].

### Pneumonia

#### Incidence and risk factors

Pneumonia is the most frequent infection in PCAS, with reported rates ranging from 45 to 75% across observational cohorts [15, 16, 20–22]. This high burden has been confirmed in multicenter observational studies, which provide the strongest available epidemiologic evidence in this field [16, 18]. Early reports described lower incidences (~ 24%), but more recent studies consistently demonstrate higher rates, likely reflecting modern ICU practices and systematic diagnostic testing [23].

Because the mechanisms of pneumonia differ substantially between arrest settings, the microbiological patterns also diverge. In OHCA, aspiration linked to impaired consciousness commonly results in infection with anaerobic organisms and enteric gram-negative bacilli. In contrast, IHCA is more frequently associated with nosocomial pathogens, such as *Pseudomonas aeruginosa*, methicillin-resistant *Staphylococcus aureus* (MRSA), and extended-spectrum β-lactamase (ESBL)—producing Enterobacterales, reflecting prolonged device exposure and prior antibiotic selection pressure. These differences highlight the need to distinguish OHCA—from IHCA-associated pneumonia when considering diagnostic evaluation and empiric antimicrobial therapy.

#### Early onset pneumonia (EOP) and VAP

EOP frequently develops within the first 48–72 h following ROSC and is most often associated with aspiration or impaired airway protection. In contrast, VAP typically emerges after prolonged mechanical ventilation and reflects ongoing exposure to nosocomial pathogens. Multiple studies have demonstrated an increased risk of both EOP and VAP in patients treated with TTM, suggesting that temperature management may contribute to susceptibility across these distinct pneumonia subtypes [15, 24].

#### Microbiology

Common pathogens in post-cardiac arrest pneumonia include both gram-negative and gram-positive organisms. Frequently isolated gram-negative bacilli include *Klebsiella* species, *Pseudomonas aeruginosa*, and *Escherichia coli*, while *Staphylococcus aureus*, including MRSA, represents a major gram-positive pathogen. Anaerobic bacteria may be encountered in EOP related to aspiration, whereas multidrug-resistant organisms are more common in patients with prolonged ICU stays or prior exposure to broad-spectrum antibiotics. These microbiological patterns should be considered when selecting empiric antimicrobial therapy in PCAS patients [18].

### UTIs

UTIs are the second most common infection type in PCAS. In a prospective OHCA cohort, Tsai et al. reported UTIs in 8% of patients within the first 7 days [20]. Mortensen et al. identified UTIs in 16% of IHCA patients vs. 6% of OHCA patients [18].

Although UTI is less common than pneumonia, it contributes to prolonged hospitalization and may provoke systemic inflammatory responses in a population already marked by immune dysregulation. The microbiological profile of UTIs in PCAS patients generally mirrors patterns observed in other critically ill populations. *Escherichia coli* remains the most common pathogen, followed by *Klebsiella* species and *Enterococcus* species, particularly in catheter-associated infections [25–27].

In IHCA patients, prolonged catheterization and extended hospitalization increase the likelihood of nosocomial pathogens, including ESBL-producing Enterobacterales and vancomycin-resistant *Enterococcus* (VRE) [28, 29]. These differences underscore the need to consider arrest setting and catheter duration when selecting empiric antimicrobial therapy and evaluating the potential for multidrug-resistant organisms.

### BSIs

BSI occurs less frequently than respiratory or urinary infections but carries significant clinical implications. Reported incidences vary widely, from 5% to as high as 38%, reflecting differences in blood-culture practices, diagnostic thresholds, and cohort characteristics. Colon Hidalgo et al. identified BSIs (reported as “bacteremia” in the original study) in 16% of patients who underwent blood cultures within the first 24 h after cardiac arrest [30]. Mortensen et al. reported an incidence of 5% in both IHCA and OHCA cohorts [18], while Tsai et al. and Gajic et al. found rates of approximately 13% and 9–12%, respectively [20, 31]. In contrast, Coba et al. documented a markedly higher incidence of 38%, a finding attributed to routine blood-culture collection in all ROSC patients and the use of broader positivity criteria [32].

The risk of BSIs appears to increase substantially with the use of mechanical circulatory support. For example, a study of OHCA patients demonstrated that those treated with extracorporeal membrane oxygenation (ECMO) had significantly higher rates of positive blood cultures compared with those managed without ECMO (39% vs. 27.7%) [33]. The predominant micro-organisms isolated in these settings are gram-positive cocci, particularly *Staphylococcus aureus* and coagulase-negative staphylococci [18, 32]. In IHCA, BSIs more commonly arise from catheter-related sources, reflecting extended exposure to central venous access devices and other invasive lines. In these patients, gram-negative pathogens such as Enterobacterales may also be isolated, particularly when prolonged hospitalization or prior broad-spectrum antibiotic exposure increases the likelihood of nosocomial infections.

Although BSIs might be expected to occur primarily in IHCA patients due to longer hospital exposure, its occurrence after OHCA is biologically plausible. Global ischemia–reperfusion injury damages the intestinal mucosa, increases epithelial permeability, and facilitates translocation of endotoxin and enteric bacteria into the bloodstream. These processes occur in parallel with post-cardiac arrest immunoparalysis, characterized by reduced monocyte HLA–DR expression and dysfunctional innate immunity [34, 35]. Supporting this mechanism, clinical studies have documented endotoxemia and elevated intestinal injury biomarkers, such as intestinal fatty acid-binding protein (I-FABP), in the early post-resuscitation period [36–38]. Given the high frequency of skin-flora contaminants in blood cultures, careful clinical adjudication, including the number of culture sets, time to positivity, and overall clinical context, is essential to distinguish true BSIs from contamination.

### Intra-abdominal infections (IAIs)

IAIs are less well-characterized in PCAS compared with respiratory or urinary infections, as only limited systematic epidemiological data exist [18]. Nevertheless, clinical experience suggests that several mechanisms may predispose patients to IAIs after cardiac arrest. Intestinal ischemia from global hypoperfusion or shock can cause epithelial injury and impair mucosal integrity [39]. Biliary stasis during prolonged critical illness and invasive procedures such as enteral feeding tube placement or percutaneous drain insertion may further contribute to infection risk [40].

At the mechanistic level, ischemia–reperfusion injury disrupts epithelial barriers through tight-junction disassembly (involving occludin, claudins, and ZO-1) and induces epithelial apoptosis. These changes increase paracellular permeability and permit translocation of endotoxin and enteric bacterial products into portal and systemic circulation [41–44]. As a result, PCAS patients may be vulnerable to infections of the gastrointestinal tract, hepatobiliary system, or peritoneal cavity, even though direct epidemiologic evidence is sparse.

Patients treated with extracorporeal cardiopulmonary resuscitation (ECPR) or ECMO appear particularly susceptible to hepatobiliary complications, including gallbladder-related infections. In a retrospective analysis, Kim et al. identified prolonged ECMO therapy as an independent risk factor for acute cholecystitis, with hemolysis considered a contributing mechanism [45].

Although IAIs are less well-studied, clinicians should maintain a high index of suspicion in PCAS patients presenting with unexplained fever, lactic acidosis, rising liver enzymes, abdominal symptoms, or hemodynamic instability. Prompt diagnostic evaluation, typically including abdominal ultrasound or computed tomography, together with targeted microbiological workup, is essential to ensure timely diagnosis and management [46].

In addition to hepatobiliary complications, post-cardiac arrest patients are also vulnerable to forms of enterocolitis arising from ischemia–reperfusion-induced gut barrier dysfunction [47, 48]. Reported entities include bacterial enteritis, *Clostridioides difficile* infection, and cytomegalovirus colitis, particularly in critically ill or immunosuppressed patients. These conditions may progress with minimal or nonspecific abdominal symptoms due to concurrent sedation or neurological impairment, underscoring the importance of maintaining clinical suspicion and pursuing early imaging and microbiological evaluation when unexplained fever, diarrhea, or hemodynamic instability occurs.

## Impact of infections on clinical outcomes after cardiac arrest

Infections significantly influence clinical outcomes in patients with PCAS, affecting mortality, neurological recovery, and the overall intensity of critical care management. These effects are mediated through complex interactions between the underlying pathophysiology of PCAS, immunosuppression, timing and type of infection, and the use of TTM. Although evidence is heterogeneous, consistent patterns emerge across cohort studies, offering important insights into the mechanisms through which infections shape prognosis following cardiac arrest.

### Impact on mortality

Several observational studies have examined the association between post-cardiac arrest infections and mortality, but findings vary considerably. Some studies have reported that pneumonia does not independently worsen survival or neurological outcomes. For example, several cohorts found no significant difference in survival to discharge or favorable neurological status between patients with and without pneumonia after cardiac arrest [15, 22, 49]. Similarly, Mongardon et al. observed comparable ICU mortality in infected and non-infected OHCA patients, suggesting that primary neurological injury often outweighs the effect of infection on overall prognosis [16].

However, the severity of infection appears to be a critical determinant of outcome. Severe infections, including sepsis, BSI, and septic shock, have consistently been associated with worse survival and organ dysfunction [9, 32]. Patients who progress to septic shock frequently experience circulatory collapse and multiple organ failure, amplifying the underlying burden of ischemia–reperfusion injury. In this context, infection acts as a major secondary insult, worsening mortality among patients who already have compromised physiological resilience.

Thus, the prognostic impact of infection in PCAS is strongly dependent on severity: mild-to-moderate infections, including uncomplicated pneumonia, do not consistently affect survival or neurological outcomes, whereas severe infections such as sepsis and septic shock represent a high-risk phenotype associated with marked physiological instability, increased mortality, and poorer neurological recovery.

### Impact on neurological recovery

Neurological outcome is a major determinant of long-term prognosis. Infections can impair neurological recovery through several mechanisms. They may exacerbate systemic inflammation—producing a “second hit” of cytokine activation—which can aggravate hypoxic–ischemic brain injury. Fever, which is common in pneumonia and BSI, is strongly associated with worse neurological outcomes and may counteract the neuroprotective effects of TTM [9]. In addition, infections often necessitate deeper or prolonged sedation, which can delay awakening and confound neurological assessment. Respiratory complications requiring reintubation or extended mechanical ventilation can further prolong time to reliable neurological evaluation [22, 49]. These effects collectively delay rehabilitation and diminish the chances of regaining functional independence.

Although not all studies demonstrate a direct causal relationship between infection and poor neurological outcome, severe infections, especially sepsis and septic shock, consistently correlate with worse neurological recovery [9, 32].

### Impact on ICU resource utilization

Infections substantially increase the burden of critical care. Multiple studies have shown that infections, including pneumonia, UTIs, and BSIs, are associated with prolonged mechanical ventilation and longer ICU and hospital stays [18, 32, 49]. These increased requirements often lead to higher rates of tracheostomy placement, further extending hospitalization and slowing recovery [22, 49].

In addition to extending hospitalization, infections contribute to secondary complications such as ICU-acquired weakness, delirium, and increased risk of subsequent infections due to prolonged immobilization and sedation [50]. Such complications can delay neurorehabilitation and complicate discharge planning, particularly in patients with limited physiological reserve [51].

Overall, infections impose significant resource demands and may prolong the path to recovery even when they do not directly influence mortality.

### Methodological variability in reported outcomes

The studies we examined marked variability regarding the prognostic significance of infection after cardiac arrest. These discrepancies arise from differences in infection definitions, timing of infection onset, patient populations (OHCA vs. IHCA), TTM protocols, and local infection-control practices [18, 52]. Some studies include only microbiologically confirmed pneumonia, whereas others rely on clinical criteria alone [15, 52]. Variation in sedation depth, neuroprognostication strategies, and decisions regarding withdrawal of life support further complicate comparisons [53, 54]. Given these inconsistencies, the impact of infection on outcomes must be interpreted with caution. However, across studies, patients with severe infections clearly experience worse outcomes, underscoring the need for early identification and proactive management [32, 55, 56].

## Pathophysiology and diagnostic challenges of infection in PCAS

### Immune dysregulation after cardiac arrest

Cardiac arrest and subsequent resuscitation induce a profound and biphasic disturbance in immune function. Much of the evidence supporting this biphasic immune response derives from mechanistic clinical studies and small prospective observational cohorts, rather than large outcome trials, which clarifies its biological relevance but limits direct extrapolation to clinical endpoints [17, 57]. Findings from these investigations consistently show that immediately after ROSC, ischemia–reperfusion injury triggers a systemic inflammatory response characterized by rapid elevation of proinflammatory cytokines, such as tumor necrosis factor-α (TNF-α) and interleukin-6 (IL-6). This early hyperinflammatory state is subsequently followed by compensatory anti-inflammatory mechanisms, resulting in cellular immune exhaustion resembling a “sepsis-like syndrome” even in the absence of infection [9, 17].Monocyte and lymphocyte dysfunction develops within hours, with ex vivo studies demonstrating markedly reduced production of TNF-α, IL-6, and interferon-γ upon lipopolysaccharide stimulation during the first several days after ROSC [58, 59]. Progressive decreases in T- and B-cell counts and impaired lymphocyte responsiveness further weaken host defenses [60, 61]. Collectively, these immune alterations increase susceptibility to secondary infections, particularly pulmonary and device-associated infections common in critical care settings.

### Immunomodulatory effects of TTM

TH, a key component of TTM, exerts immunosuppressive effects that can compromise host defense mechanisms. Hypothermia reduces leukocyte production [62], impairs neutrophil chemotaxis and phagocytosis [63], and suppresses the release of proinflammatory cytokines such as interleukin-6 (IL-6) and tumor necrosis factor-α (TNF-α) from monocytes and macrophages [64, 65]. While these effects may attenuate excessive inflammation associated with ischemia–reperfusion injury, they simultaneously impair antimicrobial host defenses. In addition, hypothermia blunts febrile responses, which can delay clinical recognition of infection [66, 67] and subsequently postpone the initiation of appropriate antimicrobial therapy.

Consistent with these mechanistic findings, observational studies have reported higher rates of pneumonia—including VAP and EOP—in patients treated with hypothermia-based TTM protocols [15, 24, 68]. Beyond aspiration and device-related factors, hypothermia-induced immune suppression is considered a contributing mechanism underlying this increased susceptibility.

More recent large randomized trials comparing TH at 33 °C with actively controlled normothermia have demonstrated differences in infection risk between these temperature-management strategies [69, 70]. TH appears to be associated with greater immunosuppression and masking of fever, potentially contributing to delayed infection detection and increased pneumonia risk. In contrast, controlled normothermia may be associated with a relatively lower infection burden. Nevertheless, immune dysfunction related to post–cardiac arrest syndrome persists even under normothermic conditions, and emerging evidence suggests that TTM in general may influence infection risk [71]. From an infection-management perspective, distinguishing TH from normothermia is, therefore, essential, and patients treated with deeper hypothermia may require enhanced surveillance and more proactive preventive strategies.

### Diagnostic challenges in identifying infection after cardiac arrest

Diagnosing infection in PCAS is challenging, because post-resuscitation physiological abnormalities mimic sepsis, and the effects of sedation and TH obscure typical clinical signs. Blunted fever responses, altered white blood cell counts, and baseline systemic inflammation limit the diagnostic value of these parameters [8, 18]. The sepsis-like inflammatory phenotype of PCAS further complicates interpretation of vital signs and laboratory markers [17, 72].

Biomarkers commonly used for infection diagnosis have limited value in the early post-cardiac arrest period. C-reactive protein (CRP) rises nonspecifically due to global ischemia–reperfusion injury, and evidence from hypothermia-treated brain-injured populations—whose pathophysiology overlaps with PCAS—suggests that CRP kinetics may be delayed or temporally altered [67]. Accordingly, early CRP values often show reduced specificity for infection in PCAS. Procalcitonin (PCT), although frequently elevated after ROSC, correlates more strongly with global injury severity than with microbiologically confirmed infection [73, 74]. Consistent with this, recent sepsis guidelines emphasize that infection cannot be reliably diagnosed on biomarkers alone [75]. Therefore, single measurements should not guide clinical decisions; interpretation must rely on serial trends integrated with clinical examination and microbiological data.

In light of these limitations, several clinical scoring systems such as the Sequential Organ Failure Assessment (SOFA) and the Clinical Pulmonary Infection Score (CPIS) may be useful as adjunctive tools for the diagnosis of infection in PCAS patients [76, 77]. Although SOFA scores are associated with hospital survival in PCAS, they are not specific to infection and may be elevated by noninfectious causes of organ dysfunction common after cardiac arrest [76]. Similarly, although CPIS was developed to support the diagnosis of VAP, several studies have not found consistent correlations between CPIS and microbiologically confirmed VAP or mortality [77]. Taken together, these findings indicate that current scoring systems, while potentially informative, lack the sensitivity and specificity required for definitive infection diagnosis in the post-cardiac arrest setting [76, 77].

Consequently, a multimodal diagnostic strategy, integrating chest imaging (chest radiography or computed tomography [CT]), targeted microbiological cultures, urinalysis, and serial biomarker trajectories with clinical assessment, is essential [17, 78, 79]. Given the non-specific nature of individual modalities, future work should prioritize the development and prospective validation of composite risk scores, which can be generated by advanced machine-learning models that integrate established physiological variables, biomarker data, and electronic health record (HER) time-series information to identify PCAS patients at high risk of infection.

## Prevention and management of post-arrest infections

Infection prevention and management in patients with PCAS require a focused and integrated strategy that accounts for the unique post-resuscitation environment. Unlike general ICU populations, PCAS patients are exposed to a convergence of factors—including neurological injury, intensive supportive care, and frequent use of invasive devices—that collectively increase infection risk and complicate timely diagnosis. Therefore, effective prevention and management strategies must integrate principles of critical care and infection control within the broader pathophysiological context of PCAS.

### General principles specific to PCAS

As detailed in Sect. "Pathophysiology and diagnostic challenges of infection in PCAS", PCAS is characterized by a biphasic immune response, with early systemic inflammation followed by profound immunosuppression, which increases susceptibility to EOP, BSIs, and device-related infections [17, 57]. In addition, TTM alters leukocyte function, cytokine responses, and fever patterns, while prolonged sedation and neuromuscular blockade limit early mobilization and airway management [64, 65, 80]. Consequently, infection control in PCAS requires enhanced surveillance, early diagnostic triggers, and proactive prevention bundles, rather than reliance on traditional clinical signs, such as fever or leukocytosis alone [17, 57, 64, 65, 80].

### Antimicrobial therapy: timing, selection, and de-escalation

Antimicrobial therapy in PCAS must account for diagnostic delays caused by sedation and TTM, which often lower the threshold for empiric treatment. Pathogens also differ by arrest setting—aspiration-related organisms are more common after OHCA, whereas IHCA is associated with nosocomial and multidrug-resistant bacteria [18]. These PCAS-specific factors should guide early empiric therapy while avoiding unnecessary broad-spectrum antibiotics.

When infection is strongly suspected—particularly pneumonia, BSI, or UTI—early empiric antimicrobial therapy is essential. Observational studies suggest that delayed antibiotic initiation may be associated with increased mortality in OHCA survivors, though confounding by survival bias must be considered [81]. Empiric therapy should be guided by the likely pathogens in PCAS: aspiration-related anaerobes in OHCA, gram-negative bacilli (*Klebsiella* spp., *Pseudomonas aeruginosa*, *Escherichia coli*), and *Staphylococcus aureus,* including MRSA [18, 20, 82]. Selection of initial therapy should reflect local resistance patterns, prior antibiotic exposure, and the patient's hospitalization history [83].

Recent RCT data in patients with acute brain injury (PROPHY–VAP trial) demonstrated that a single prophylactic 2 g dose of ceftriaxone administered within 12 h of intubation reduced early VAP (days 2–7) from 32 to 14% [84]. Although PCAS shares some pathophysiological overlap with acute brain injury, extrapolation requires caution, and any adoption should balance potential benefits against stewardship considerations.

Routine prophylactic broad-spectrum antibiotics are not recommended because of risks involving antimicrobial resistance and Clostridioides difficile infection [85, 86]. Once microbiological data are available, rapid de-escalation to narrower-spectrum agents is crucial to minimize toxicity and selection pressure [87, 88]. In patients with ambiguous signs due to TTM or sedation, serial CRP or PCT measurements may assist in guiding therapy duration, although their diagnostic specificity is limited in PCAS and interpretations require caution [49, 89].

### Prevention bundles and nonpharmacologic measures

Given the high burden of device exposure after cardiac arrest, the implementation of structured prevention bundles is fundamental to effective infection control. These bundles emphasize several core practices, beginning with strict adherence to hand hygiene and aseptic technique, both of which represent the cornerstone of preventing device-associated infections. Elevating the head of the bed to at least 30° is recommended to mitigate aspiration risk, particularly in patients who are mechanically ventilated or have impaired airway protection. Sedation strategies should incorporate daily interruption and spontaneous awakening or breathing trials, which facilitate early assessment, reduce ventilator duration, and thereby lower the incidence of ventilator-associated complications. The timely removal of urinary catheters and unnecessary central venous lines is equally important, as prolonged device use is a well-established risk factor for CAUTIs and CRBSIs. In addition, structured oral hygiene protocols—including toothbrushing, suctioning, and antiseptic use according to institutional policy—play a significant role in reducing oropharyngeal colonization and subsequent ventilator-associated pneumonia [90, 91].

Beyond these core measures, more targeted approaches such as selective digestive decontamination (SDD) and selective oropharyngeal decontamination (SOD) have demonstrated efficacy in reducing pneumonia in certain intensive care settings [92]. However, their widespread adoption remains controversial due to concerns about promoting antimicrobial resistance and the feasibility of implementation across diverse institutions [86].

### Airway management and aspiration prevention

Preventing aspiration, particularly in OHCA patients, is a key component of early infection control. Rapid airway protection during resuscitation and meticulous airway management in the ICU are essential [9, 93]. Supraglottic airway devices have not been conclusively shown to increase EOP, supporting their reasonable use depending on provider expertise and prehospital context [49, 90]. During mechanical ventilation, maintaining appropriate cuff pressures, minimizing unnecessary sedation, and pursuing early extubation when appropriate reduce the risk of VAP [94]. Airway care should follow established VAP bundle principles [89].

### Nutrition, glycemic control, and sedation management

Nonpharmacologic critical care strategies play an important role in reducing infection risk. Early enteral nutrition supports gut barrier integrity and may prevent bacterial translocation, potentially lowering risk for BSIs [95, 96]. In critically ill patients, enteral feeding has been associated with reduced nosocomial infections, including pneumonia and sepsis [97]. Evidence from PCAS-specific cohorts indicates that early enteral feeding is safe and not associated with increased infection risk [98].

Hyperglycemia, especially during TTM, has been linked to increased infections and worse neurological outcomes [99, 100]. Maintaining glucose within target ranges may, therefore, indirectly reduce infection complications.

Minimizing deep sedation facilitates early awakening, mobility, and ventilator liberation, helping reduce VAP and other ICU-related complications [94, 101, 102]. This is particularly important in PCAS, where sedation delays neuroprognostication and increases device exposure time.

## Future directions and research gaps

**Fig. 1:**
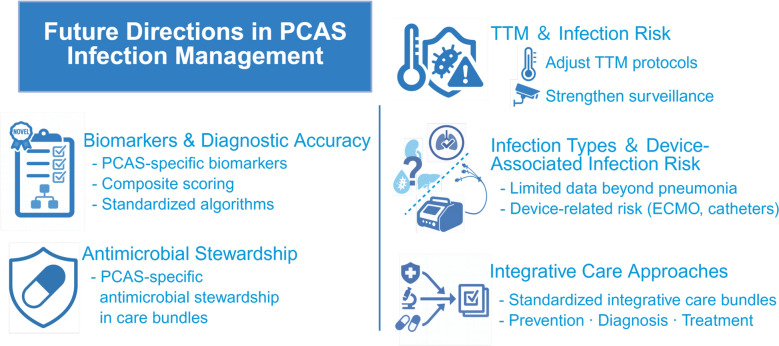
Future directions for infection management in post–cardiac arrest syndrome

Although awareness of the importance of infection management in PCAS is increasing, substantial evidence gaps still exist, hindering the implementation of timely and effective treatment. To advance future care, a shift is needed from fragmented approaches to a comprehensive and protocol-driven infection management system within PCAS care. Table 2 outlines key research gaps and highlights how progress in diagnostics, antimicrobial stewardship, and integrated care pathways may translate into more consistent and clinically effective infection management. Priority areas for future investigation include the following: quantifying the infection-attributable burden of morbidity and mortality; the development and validation of diagnostic biomarkers and composite clinical decision tools—including machine-learning early warning models that leverage EHR data; antibiotic use strategies, including evaluation of time-limited prophylactic antibiotics immediately after intubation in selected high-risk patients; TTM-related immunomodulation; under-recognized infection types and device-associated risk; and system-level integration into standardized care pathways (Fig. 1).

**Table 2:** Research gaps and future directions in infection management after cardiac arrest

Research area	Challenges/gaps	Future directions	Clinical implications
Biomarkers and diagnostic accuracy	Low specificity of CRP/PCT due to reperfusion injury [73, 74, 103–105]	Identify PCAS-specific markers or composite scores; standardize protocols [74, 104]	Improved diagnostic precision may reduce unnecessary antibiotic exposure, support earlier targeted therapy, and prevent delays in infection recognition during TTM
Antimicrobial stewardship	Unclear timing/duration of antibiotics, esp. for EOP [15, 16, 22, 88, 110]	Develop stewardship protocols; assess prophylactic strategies (e.g., PROTECT trial [111])	Optimized antibiotic use reduces resistance, decreases C. difficile risk, and ensures time-sensitive initiation while avoiding overtreatment
Temperature management and infection risk	TTM masks infection signs and suppresses immunity [13–15, 24, 64, 68, 119]	Adjust TTM protocols; embed infection prevention in TTM care [19]	Better alignment of TTM practices with infection prevention may reduce aspiration events, improve fever detection, and enhance early diagnosis
Infection types and device-associated risk	Non-pulmonary infections are under-studied, often device-related, and frequently detected late [20, 32, 45, 115, 116]	Study non-pulmonary and device-related infections; define risks and outcomes, and develop prevention for high-risk patients	Earlier recognition of BSI, CAUTI, and IAIs may reduce septic progression, guide earlier imaging and culture strategies, and improve outcomes in ECMO/ECPR patients
Integrative care approaches	Infection management remains fragmented [1, 9, 10, 16, 86, 117]	Create standardized care bundles for infection control [1, 9, 86]	Unified ICU pathways reduce practice variability, support consistent surveillance, and optimize neurological and survival outcomes in PCAS

CRP: C-reactive protein; PCT: procalcitonin; PCAS: post-cardiac arrest syndrome; EOP: early onset pneumonia; TTM: targeted temperature management; BSI: bloodstream infection; CAUTI: catheter-associated urinary tract infection; IAI: intra-abdominal infection; ECMO: extracorporeal membrane oxygenation; ECPR: extracorporeal cardiopulmonary resuscitation

### Biomarkers and diagnostic accuracy

A central research need is the improvement of diagnostic precision. Traditional biomarkers such as CRP and PCT show reduced specificity after cardiac arrest, because ischemia–reperfusion injury elevates these markers irrespective of infection, thereby limiting their diagnostic accuracy [17, 73, 74, 103–105]. Novel diagnostic tools, particularly multiplex PCR respiratory pathogen panels, have shown promise in ICU settings by enabling rapid, comprehensive pathogen identification and facilitating early de-escalation of antimicrobials [106]. In addition, advances in host-response transcriptomics [107], immune-phenotyping approaches, including single-cell transcriptomic profiling [108], and machine-learning prediction models using EHR-derived time-series data [109] may offer earlier and more accurate identification of infection phenotypes, although these tools require prospective validation in PCAS-specific cohorts. Composite scoring systems integrating dynamic biomarker trajectories, bedside examination, and imaging findings also warrant development and validation to enhance diagnostic accuracy in the TTM era.

### Antimicrobial stewardship

Evidence guiding the appropriate timing, selection, and duration of antibiotic therapy in patients with PCAS remains limited [15, 16, 22, 88, 110]. In particular, for EOP, it remains unclear whether empiric therapy improves clinical outcomes or leads to unnecessary antibiotic exposure. The ongoing PROTECT trial aims to evaluate prophylactic ceftriaxone in comatose OHCA survivors [111]. Across ICU populations, PCT-guided discontinuation strategies—triggered by sustained decreases or prespecified thresholds—have been externally validated to safely shorten antibiotic courses [112, 113]. In PCAS, any PCT-guided approach should emphasize serial trajectories rather than single timepoints and be integrated with clinical and microbiological assessment, given ischemia–reperfusion-related elevations. Incorporating the principles of antimicrobial stewardship into PCAS-specific care bundles may help reduce inappropriate antibiotic use, prevent the emergence of resistant organisms, and ultimately improve clinical outcomes. Future work should also assess time-limited post-intubation prophylaxis in carefully selected high-risk patients within stewardship frameworks.

### Temperature management and infection risk

As discussed in Sect. "Pathophysiology and diagnostic challenges of infection in PCAS", TTM, including TH, has been linked to increased infection risk and masking of typical clinical signs, based on single-center observational studies and systematic reviews [15, 24]. Building on this background, emerging data suggest that infection risk may also vary according to specific TTM parameters. Lower target temperatures (e.g., 33 °C) have been associated with higher rates of pneumonia and prolonged ventilation requirements, while slower rewarming may impair immune cell function and delay the detection of fever [15, 114]. These observations highlight the need to clarify how individual components of TTM—such as target temperature, maintenance duration, and rewarming speed—modulate infection risk without compromising neuroprotection.

Future investigations should, therefore, move beyond descriptive epidemiology and systematically evaluate whether protocol modifications can reduce infection risk in PCAS populations. In addition, systems for infection surveillance, prevention, and early intervention should be incorporated in advance into the standard flow of TTM care [19].

### Infection types and device-associated risk

Non-pulmonary infections in PCAS, such as UTIs, BSIs, and IAIs, remain insufficiently studied. These infections may progress asymptomatically, leading to delayed detection [20, 32, 45, 115, 116]. The incidence, risk factors, and outcomes in patients undergoing ECMO or those with long-term use of invasive devices should be systematically evaluated. Understanding these infection profiles, particularly in high-risk populations, is essential for informing the development of integrative care strategies.

### Integrative care approaches

To optimize outcomes, prevention, diagnosis, and treatment of infections should be incorporated into standardized care bundles. These bundles should include early risk assessment, diagnostic triggers, biomarker-guided antibiotic use, and monitoring of device-related infections [1, 9, 10, 16, 86, 117]. The development and implementation of such integrated care pathways are expected to enhance consistency, reduce practice variability, and strengthen clinical decision-making across diverse ICUs [1, 9, 86]. Ultimately, a shift toward precision medicine guided by biomarker phenotypes, individualized infection-risk stratification, and personalized TTM profiles may offer the most meaningful improvements in survival and neurological recovery for patients with PCAS.

## Limitations

This narrative review has several limitations. First, because it relied primarily on observational studies, single-center cohorts, and heterogeneous diagnostic definitions, the reported infection rates and outcome associations must be interpreted with caution. Second, much of the historical literature was conducted in the era of TH rather than contemporary TTM practices, making it difficult to fully extrapolate older findings to current clinical care. Third, diagnostic criteria for pneumonia, BSIs, and intra-abdominal infections varied substantially across studies, which limited direct comparison and may have introduced classification bias. Fourth, because narrative synthesis does not include a formal risk-of-bias assessment or meta-analytic weighting, the conclusions may be influenced by publication bias and differences in study methodology. Finally, emerging areas such as machine-learning decision tools, PCAS-specific diagnostic biomarkers, and targeted antimicrobial stewardship strategies remain insufficiently studied, underscoring the need for prospective, multicenter research to strengthen future evidence.

## Conclusion

Infections are highly prevalent among patients with PCAS, with pneumonia representing the most common complication. These infections frequently prolong ICU stay, increase the need for mechanical ventilation, and delay overall recovery, especially in the setting of sedation practices and TTM. Although mild-to-moderate infections may not consistently predict poor neurological outcomes on their own, progression to advanced sepsis with organ dysfunction or septic shock clearly worsens physiological instability and can adversely affect both survival and neurological recovery.

Diagnostic challenges in PCAS arise not only from TTM-induced immunosuppression and blunting of typical clinical signs, but also from the systemic inflammatory response and organ dysfunction that occur after cardiac arrest itself. Because these factors reduce the reliability of conventional indicators, such as fever and leukocytosis, a multimodal diagnostic approach that integrates imaging, microbiological testing, and serial biomarker trends is required.

From a clinical perspective, incorporating structured infection surveillance, early diagnostic triggers, and stewardship-based antimicrobial strategies into standard post-cardiac arrest care may help reduce preventable complications and variability across ICUs. Embedding these practices within TTM workflows, where clinical signs are frequently obscured, has the potential to improve early detection and optimize neurological and survival outcomes.

Interpretation of the existing evidence requires caution, because studies vary substantially in infection definitions, diagnostic practices, TTM protocols, and illness–severity adjustment. Most available data come from observational cohorts with heterogeneous methodologies, making direct comparison challenging. Nevertheless, the consistent observation of high pneumonia burden and the clear impact of severe infections across studies support the clinical relevance of structured surveillance and standardized infection management in PCAS.

Future research should aim to refine diagnostic specificity, quantify the burden of infectious complications, and clarify how the severity and progression of infection influence neurological and survival outcomes. Until these gaps are addressed, integrated and proactive infection control remains an essential element of optimizing care and improving outcomes for patients with PCAS.

## Data Availability

No datasets were generated or analysed during the current study.
